# Risk Factors Associated With Major Cardiovascular Events 1 Year After Acute Myocardial Infarction

**DOI:** 10.1001/jamanetworkopen.2018.1079

**Published:** 2018-08-10

**Authors:** Yun Wang, Jing Li, Xin Zheng, Zihan Jiang, Shuang Hu, Rishi K. Wadhera, Xueke Bai, Jiapeng Lu, Qianying Wang, Yetong Li, Chaoqun Wu, Chao Xing, Sharon-Lise Normand, Harlan M. Krumholz, Lixin Jiang

**Affiliations:** 1 Department of Biostatistics, Harvard T.H. Chan School of Public Health, Boston, Massachusetts; 2 Center for Outcomes Research and Evaluation, Yale-New Haven Hospital, New Haven, Connecticut; 3 Richard and Susan Smith Center for Outcomes Research in Cardiology, Division of Cardiology, Beth Israel Deaconess Medical, Harvard Medical School, Boston, Massachusetts; 4 National Clinical Research Center of Cardiovascular Diseases, State Key Laboratory of Cardiovascular Disease, Fuwai Hospital, National Center for Cardiovascular Diseases, Chinese Academy of Medical Sciences and Peking Union Medical College, Beijing, China; 5 Department of Cardiology, Peking University People’s Hospital, Beijing Key Laboratory of Early Prediction and Intervention of Acute Myocardial Infarction, Beijing, China; 6 Brigham and Women’s Hospital Heart & Vascular Center, Harvard Medical School, Boston, Massachusetts; 7 Department of Health Care Policy, Harvard Medical School, Boston, Massachusetts; 8 Section of Cardiovascular Medicine, Department of Internal Medicine, Yale School of Medicine, New Haven, Connecticut; 9 Department of Health Policy and Management, Yale School of Public Health, New Haven, Connecticut

## Abstract

**Importance:**

Patients who survive acute myocardial infarction (AMI) have a high risk of subsequent major cardiovascular events. Efforts to identify risk factors for recurrence have primarily focused on the period immediately following AMI admission.

**Objectives:**

To identify risk factors and develop and evaluate a risk model that predicts 1-year cardiovascular events after AMI.

**Design, Setting, and Participants:**

Prospective cohort study. Patients with AMI (n = 4227), aged 18 years or older, discharged alive from 53 acute-care hospitals across China from January 1, 2013, to July 17, 2014. Patients were randomly divided into samples: training (50% [2113 patients]), test (25% [1057 patients]), and validation (25% [1057 patients]). Risk factors were identified by a Cox model with Markov chain Monte Carlo simulation and further evaluated by latent class analysis. Analyses were conducted from May 1, 2017, to January 21, 2018.

**Main Outcomes and Measures:**

Major cardiovascular events, including recurrent AMI, stroke, heart failure, and death, within 1 year after discharge for the index AMI hospitalization.

**Results:**

The mean (SD) age of the cohort was 60.8 (11.8) years and 994 of 4227 patients (23.5%) were female. Common comorbidities included hypertension (2358 patients [55.8%]), coronary heart disease (1798 patients [42.5%]), and dyslipidemia (1290 patients [30.5%]). One-year event rates were 8.1% (95% CI, 6.91%-9.24%), 9.0% (95% CI, 7.22%-10.70%), and 6.4% (95% CI, 4.89%-7.85%) for the training, test, and validation samples, respectively. Nineteen risk factors comprising 15 unique variables (age, education, prior AMI, prior ventricular tachycardia or fibrillation, hypertension, angina, prearrival medical assistance, >4 hours from onset of symptoms to admission, ejection fraction, renal dysfunction, heart rate, systolic blood pressure, white blood cell count, blood glucose, and in-hospital complications) were identified. In the training, test, and validation samples, respectively, the risk model had C statistics of 0.79 (95% CI, 0.75-0.83), 0.73 (95% CI, 0.68-0.78), and 0.77 (95% CI, 0.70-0.83) and a predictive range of 1.2% to 33.9%, 1.2% to 37.9%, and 1.3% to 34.3%. The C statistic was 0.69 (95% CI, 0.65-0.74) for the latent class model in the training data. The risk model stratified 11.3%, 81.0%, and 7.7% of patients to high-, average-, and low-risk groups, with respective probabilities of 0.32, 0.06, and 0.01 for 1-year events.

**Conclusions and Relevance:**

Nineteen risk factors were identified, and a model was developed and evaluated to predict risk of 1-year cardiovascular events after AMI. This may aid clinicians in identifying high-risk patients who would benefit most from intensive follow-up and aggressive risk factor reduction.

## Key Points

**Question:**

What are the risk factors associated with major cardiovascular events within 1 year among patients who survive an acute myocardial infarction?

**Findings:**

In this cohort study of 4227 patients with acute myocardial infarction, 19 risk factors associated with major cardiovascular events 1 year after acute myocardial infarction, comprising 15 unique variables, were identified. A risk model to predict 1-year cardiovascular events after an acute myocardial infarction was developed and evaluated based on these risk factors.

**Meaning:**

Identification of individuals at the highest risk of long-term cardiovascular events after acute myocardial infarction may aid in the provision of targeted, intensive, and higher-quality longitudinal care following discharge.

## Teaser

This cohort study identifies risk factors associated with major cardiovascular events within 1 year of acute myocardial infarction in Chinese patients and describes the development and evaluation of a risk model to predict such events.

## Introduction

Patients who survive an acute myocardial infarction (AMI) are at high risk of major cardiovascular events following discharge, although risk may vary considerably between patients. Lifestyle changes, quality of care, and treatment with guideline-directed medical therapy after AMI are all important for secondary prevention. Patient characteristics are also associated with risk of cardiovascular events. Identification of these characteristics would better inform patients, families, and physicians about the risk of cardiovascular events following discharge and help ensure intensive follow-up and risk factor modification. Efforts to identify risk factors have focused on the period immediately after initial hospitalization for AMI and have not provided a longer-term perspective. Moreover, studies on long-term outcomes after an AMI predominately focus on mortality.^1,2,3,4^ Major cardiovascular events following discharge, such as recurrent AMI, stroke, and heart failure, all adversely affect a patient’s quality of life and are, in aggregate, much more common than death.^1,5,6,7,8^ There is a need to focus on long-term outcomes beyond mortality to capture the complete health care experience of patients, particularly in non-Western countries.

Accordingly, we used the *China Patient-Centered Evaluative Assessment of Cardiac Events Prospective Study of AMI* (China PEACE Prospective AMI study)^9^ data to identify clinically important risk factors and develop and evaluate a risk model to predict major cardiovascular events up to 1 year following discharge for AMI. This study, which includes data abstracted from medical records and detailed longitudinal information about patients with AMI, is ideally positioned to generate tools to further stratify risk in the year after hospital discharge.

## Methods

### Study Sample

The China PEACE Prospective AMI study consecutively enrolled 5901 patients with AMI aged 18 years or older who were discharged alive from acute-care hospitals in China from January 1, 2013, through July 17, 2014.^9^ Patient information was collected during the index AMI hospitalization and at 1, 6, and 12 months following discharge. Medical records were abstracted independently by 2 trained abstractors; agreement was 98% for key data elements. During the 1-year follow-up period, trained staff interviewed patients and systematically collected information on all clinical events, including recurrent AMI, angina, ischemic or hemorrhagic stroke, heart failure, transient ischemic attack, repeated angiography procedures, revascularization, bleeding, and rehospitalizations. Death information was obtained from the family or physician for patients who died during follow-up. For patients who were unable to attend an onsite interview, information was obtained by telephone through direct correspondence with the patient, the patient’s family members, or the patient’s physician.

We excluded patients who were transferred to another acute-care hospital (n = 113), died prior to discharge (n = 287), did not agree to participate in the follow-up interviews (n = 1206), or were lost to follow-up immediately after discharge (n = 68). The final sample included 4227 unique patients who we randomly divided into 3 mutually exclusive samples for training (50% [2113 patients]), test (25% [1057 patients]), and validation (25% [1057 patients]) analyses. The training sample was used to select risk factors; test and validation samples were used for subsequent evaluation. Our rationale for creating the test and evaluation samples, rather than just 1 test sample, was to gain an additional opportunity to assess the stability and performance of the risk model. The Human Investigation Committee of China’s National Clinical Research Center of Cardiovascular Diseases approved this study. All patients provided written informed consent. The study followed the Transparent Reporting of a Multivariable Prediction Model for Individual Prognosis or Diagnosis (TRIPOD) reporting guideline.^10^

### Potential Risk Factors

We selected candidate risk factors that were clinically meaningful, were supported by the literature, were reliable and easily collected, occurred at a frequency of more than 1%, and were present before or during the index AMI hospitalization. Initial factors included patient demographic characteristics (age, sex, education, occupation, insurance), medical history (hypertension, diabetes mellitus, angina, coronary heart disease, prior ventricular tachycardia or ventricular fibrillation, atrial fibrillation, heart failure, prior AMI, prior percutaneous coronary intervention, prior coronary artery bypass grafting), lifestyle factors (smoking, drinking, snoring during natural sleep), time between onset of symptoms and admission greater than 4 hours, prearrival medical assistance, in-hospital diagnoses (ST segment elevation, AMI inferior or anterior, Killip class 3 or 4, ejection fraction [EF] <40%), renal dysfunction (blood urea nitrogen >40 mg/dL [to convert to mmol/L, multiply by 0.357] or creatinine >2.5 mg/dL [to convert to μmol/L, multiply by 88.4]), admission temperature (°C), heart rate greater than 90 beats per minute, blood glucose greater than 216 mg/dL (to convert to mmol/L, multiply by 0.0555), systolic blood pressure greater than 100 mm Hg, white blood cell count (WBC) 6000 to 12 000 per μL or greater than 12 000 per μL (to convert to ×10^9^/L, multiply by 0.001), in-hospital treatments (coronary artery bypass grafting, percutaneous coronary intervention, medications), and number of in-hospital complications (recurrent angina, recurrent AMI, pericardial effusion, atrial fibrillation, cardiopulmonary resuscitation, ventricular tachycardia or fibrillation, new heart failure, infection, stroke, major bleeding) rated for severity on a scale of 0 to 10, with 10 being the most severe. Factors with missing values were imputed using multiple imputation with 10 imputations.^11^ The final imputed value was an average of the 10 imputations. Rates of missing ranged from 0.2% (systolic blood pressure and WBC) to 2.7% (blood glucose).

### Outcome

The outcome for the prediction model was 1-year major cardiovascular events, a binary variable defined as the occurrence of recurrent AMI, stroke (ischemic or hemorrhagic), heart failure, or death within 1 year of discharge for the index AMI hospitalization. Information on the outcome was obtained through interviews and confirmed using medical records (eAppendix 1 in the Supplement).

### Statistical Analysis

#### Risk Factor Selection and Evaluation

Using the training sample with all candidate risk factors, we first fit a stepwise Cox proportional hazards model to eliminate factors that were unlikely to predict the outcome, with a *P* value threshold of .35 for adding factors and .25 for removing factors. All *P* values were 2-sided. Next, we fit the Cox model with Markov chain Monte Carlo (MCMC) simulation and calculated a posterior probability for each factor selected by the stepwise procedure.^12^ The posterior probability measures the strength of an association between a factor and the outcome. A factor with a posterior probability greater than 0.95 (or <0.05 for factors with estimates <0.0) was considered significant for predicting the outcome and therefore included in the final risk factor list.^13,14^ We then constructed the final risk model to predict 1-year events by refitting the Cox model to the training sample, without stepwise elimination, using the selected risk factors.

We calculated the following indicators to evaluate the risk model performance: the Harrell C statistic to assess the overall predictive accuracy,^15^ time-dependent area under the receiver operating characteristic (ROC) curves to assess the predictive accuracy over the entire 1-year period,^16^ the Schemper-Henderson measure to assess explained variation,^17^ the Hosmer-Lemeshow goodness-of-fit test to assess calibration, and Schoenfeld residuals to assess the proportional hazards assumption.^18^ Discrimination was assessed among the observed outcomes in strata defined by deciles of the predictive probabilities.^19^ We divided patients in the training sample into 10 mutually exclusive risk classes based on these deciles, ranking the classes from lowest risk (class 1) to highest risk (class 10) for evaluation.

To further evaluate the selected risk factors, we used the training data to conduct a latent class analysis, which uses an unsupervised machine learning algorithm that does not require an outcome. Conceptually, if the selected risk factors are substantially associated with the outcome, they will have the ability to assign patients to risk classes with unsupervised learning algorithms. Thus, we used latent class analysis to classify patients into 10 mutually exclusive classes and ranked them from lowest risk (class 1) to highest risk (class 10) on the basis of the observed outcome. We chose 10 classes to align with the decile-specific classes based on the risk model described previously. We calculated a Spearman correlation coefficient between the risk classes based on the risk model and the risk classes based on latent class analysis. A high coefficient indicates good agreement between the 2 classified results, which provides information on the robustness of the selected risk factors. We further validated the risk model by comparing its performance in the training sample with its performance in the test and validation samples (eAppendix 2 in the Supplement).

#### Risk Score

To facilitate the use of the selected risk factors and the risk model, we constructed a simple risk score for each patient based on the regression coefficients estimated from the risk model with the training sample. Points for each risk factor were calculated by dividing the risk factor’s coefficient by the sum of all coefficients in the risk model, multiplying by 100, and rounding to the nearest integer. We then calculated a risk score for each patient by summing that patient’s points.^20,21^ We stratified patients into 3 risk groups based on the distribution of the risk score: low (<10th percentile), average (10th-90th percentile), and high (>90th percentile). We further generated Kaplan-Meier survival curves to illustrate the probability of being free from 1-year events by the 3 risk groups.

Analyses were conducted between May 1, 2017, and January 21, 2018, using SAS statistical software version 9.4 (SAS Institute Inc). Latent class analysis was conducted using the PROC LCA procedure, version 1.3.2 beta.^22^

## Results

### Study Sample

A total of 4227 (2113 training, 1057 test, and 1057 validation) patients across 53 hospitals were included. The mean (SD) age was 60.8 (11.8) years, and 994 patients (23.5%) were female (eTable 1 in the Supplement). The most common comorbidities were hypertension (2358 patients [55.8%]), coronary heart disease (1798 patients [42.5%]), and dyslipidemia (1290 patients [30.5%]). Patient characteristics across the training, test, and validation samples were similar (eTable 1 in the Supplement). Median (interquartile range [IQR]) length of hospital stay was 11 (8-14) days.

### One-Year Cardiovascular Events

For the training, test, and validation samples, respectively, the rates of 1-year cardiovascular events were 8.1% (95% CI, 6.91%-9.24%; 171 of 2113 patients), 9.0% (95% CI, 7.22%-10.70%; 94 of 1057 patients), and 6.4% (95% CI, 4.89%-7.85%; 67 of 1057 patients) (*P* = .36), and the number of cardiovascular events per patient ranged from 1 to 6, 1 to 7, and 1 to 6. Approximately 79.2% of patients (263 of 332) had 1 event, and 15.7% (52 of 332) had 2 events. The most common event was heart failure (249 of 4227 patients [5.9%]), followed by recurrent AMI (139 of 4227 patients [3.3%]) and stroke (42 of 4227 patients [1.0%]). In 4227 patients, the nonfatal cardiovascular event rate was 5.1% (216 patients). The 1-year cardiovascular-related and non–cardiovascular-related mortality rates were 2.4% (101 patients) and 0.3% (13 patients), respectively (total mortality rate, 2.7% [114 patients]). The median (IQR) time from discharge to an event was 89 (35-207) days; recurrent AMI occurred earlier than other events (median [IQR], 78 [10-198] days) (eFigure 1 in the Supplement). The individual events that made up the composite cardiovascular event outcome were positively correlated, with the tetrachoric correlation coefficient ranging from 0.06 (stroke and heart failure) to 0.57 (AMI and death).

### Risk Factor Selection and Validation

The stepwise Cox regression identified 23 candidate factors, and MCMC simulation removed 4 that had a posterior probability of less than 0.95. The remaining 19 risk factors (Table 1), represented by 15 unique variables, include patient demographics (age, college degree), comorbidities (prior AMI, prior ventricular tachycardia or fibrillation, hypertension, angina), hospital diagnoses and tests (EF <40%, EF unable to measure, renal dysfunction, heart rate >90 beats per minute, blood glucose >216 mg/dL, SBP <100 mm Hg, WBC 6000/μL-12 000/μL, WBC >12 000/μL), access to care (prearrival medical assistance, time from onset of symptoms to admission >4 hours), and in-hospital complications (eFigure 2 in the Supplement). The risk model based on the 19 risk factors and the training sample demonstrated good discrimination, calibration, and fit. For the risk model, the overall C statistic was 0.79 (95% CI, 0.75-0.83), and the time-dependent area under the ROC curve ranged from 0.79 (95% CI, 0.75-0.83) to 0.75 (95% CI, 0.71-0.79) (eFigure 3 in the Supplement). The mean observed 1-year outcome ranged from 1.2% in the lowest predicted decile to 33.9% in the highest predicted decile, a range of 32.7% (eFigure 4 in the Supplement). The *P* value of the Hosmer-Lemeshow goodness-of-fit test was .77, suggesting the model was fitted well (eFigure 5 in the Supplement). The explained variation was 0.14, and the Schoenfeld residuals test indicated that all risk factors, except for WBC greater than 12 000/μL and renal dysfunction, met the proportional hazards assumption.

**Table 1.** Model-Selected Patient Risk Factors by Training, Test, and Validation Samples

Risk Factor (n = 19)	No. (%)			
	Aggregate (N = 4227)	Training (n = 2113)	Test (n = 1057)	Validation (n = 1057)
Age, y				
65-74	1068 (25.3)	530 (25.1)	241 (22.8)	297 (28.1)
75-84	536 (12.7)	263 (12.4)	133 (12.6)	140 (13.3)
≥85	51 (1.2)	22 (1.0)	18 (1.7)	11 (1.0)
No college degree	3661 (86.6)	1822 (86.2)	918 (86.9)	921 (87.1)
No prearrival medical assistance	2655 (62.8)	1296 (61.3)	690 (65.3)	669 (63.3)
Prior angina	166 (3.9)	86 (4.1)	43 (4.1)	37 (3.5)
Prior acute myocardial infarction	332 (7.9)	164 (7.8)	83 (7.9)	85 (8.0)
Prior ventricular tachycardia or ventricular fibrillation	100 (2.4)	46 (2.2)	23 (2.2)	31 (2.9)
Hypertension	2358 (55.8)	1164 (55.1)	590 (55.8)	604 (57.1)
Symptoms to admission >4 h	2438 (57.7)	1227 (58.1)	617 (58.4)	594 (56.2)
Renal dysfunction (blood urea nitrogen >40 mg/dL or creatinine >2.5 mg/dL)	844 (20.0)	401 (19.0)	239 (22.6)	204 (19.3)
Ejection fraction value				
<40%	310 (7.3)	149 (7.1)	86 (8.1)	75 (7.1)
Unmeasured	598 (14.1)	293 (13.9)	159 (15.0)	146 (13.8)
White blood cell count				
6000/μL-12 000/μL	2882 (68.2)	1439 (68.1)	724 (68.5)	719 (68.0)
>12 000/μL	351 (8.3)	191 (9.0)	85 (8.0)	75 (7.1)
Fasting blood glucose >216 mg/dL	254 (6.0)	125 (5.9)	68 (6.4)	61 (5.8)
Heart rate >90 beats/min	598 (14.1)	307 (14.5)	139 (13.2)	152 (14.4)
Systolic blood pressure <100 mm Hg	332 (7.9)	167 (7.9)	80 (7.6)	85 (8.0)
No. of in-hospital complications, mean (SD)	0.85 (1.0)	0.84 (1.0)	0.89 (1.0)	0.81 (1.0)

SI conversion factors: To convert blood urea nitrogen to mmol/L, multiply by 0.357; creatinine to μmol/L, multiply by 88.4; white blood cell count to ×10^9^/L, multiply by 0.001; and blood glucose to mmol/L, multiply by 0.0555.

The latent class analysis assigned each of the 2113 patients in the training sample into 10 classes based on the combination of 19 risk factors (eFigure 6 in the Supplement). The area under the ROC curve for this analysis was 0.69 (95% CI, 0.65-0.74), and the mean observed outcome rate ranged from 3.4% in the lowest rating group to 19.3% in the highest rating group, a range of 15.9%. The Spearman correlation coefficient between the predicted decile based on the Cox risk model and the latent class analysis was 0.60 (95% CI, 0.57-0.63).

The probabilities of 1-year events based on regression coefficients estimated from the risk model in the training sample and the risk model in the test and validation samples were highly correlated (eFigure 7 in the Supplement). Model performance in test and validation samples was comparable to that in the training sample. For the test and validation samples, respectively, the overall C statistic was 0.73 (95% CI, 0.68-0.78) and 0.77 (95% CI, 0.70-0.83); the rate of observed 1-year events ranged from 1.2% and 1.3% in the lowest predicted decile to 37.9% and 34.3% in the highest predicted decile; the explained variations were 0.09 and 0.16.

### Risk Score

**Figure 1.**
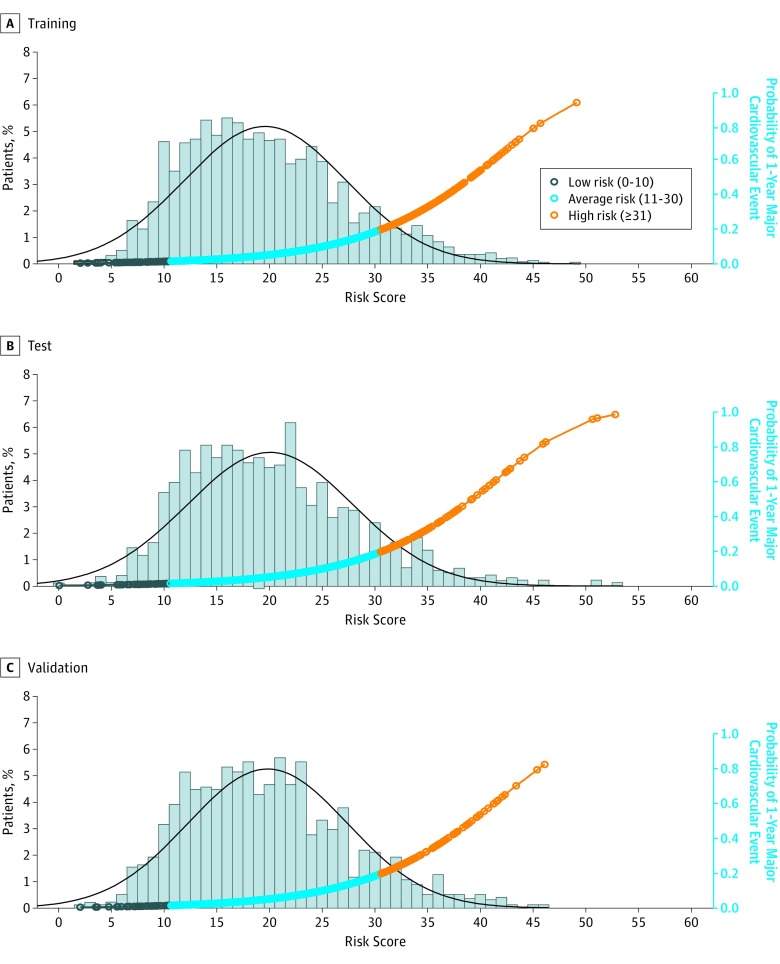
Distribution of Patient Risk Scores and Probability of 1-Year Major Cardiovascular Events by Risk Score The histograms show the distribution of risk scores for training (A), test (B), and validation (C) samples, and the curves show the probability of major cardiovascular events at 1 year. The black curve represents a fitted density curve on the risk score histogram. A risk score, ranging from 0 to 100, was constructed at the patient level based on the regression coefficients estimated from the final risk model with the training sample. A higher risk score indicates a higher probability of major cardiovascular events 1 year after discharge.

**Figure 2.**
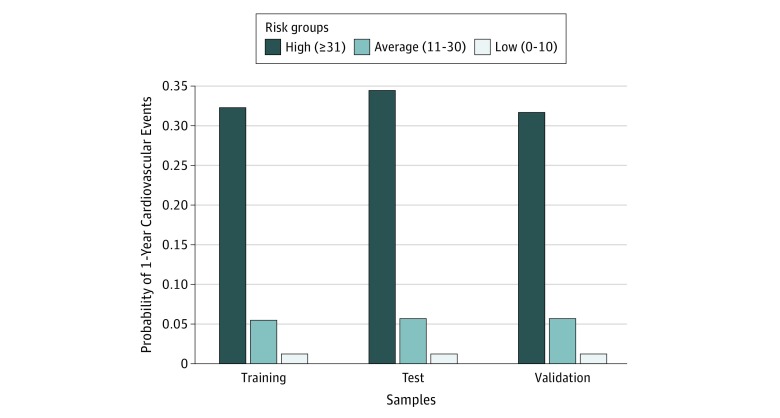
Risk Stratification by Risk Scores For the training, test, and validation samples, respectively, the highest risk group includes 11.3%, 12.1%, and 11.7% of the patients; the average risk group includes 81.0%, 81.8%, and 81.1% of the patients; and the lowest risk group includes 7.7%, 6.1%, and 7.2% of the patients.

**Figure 3.**
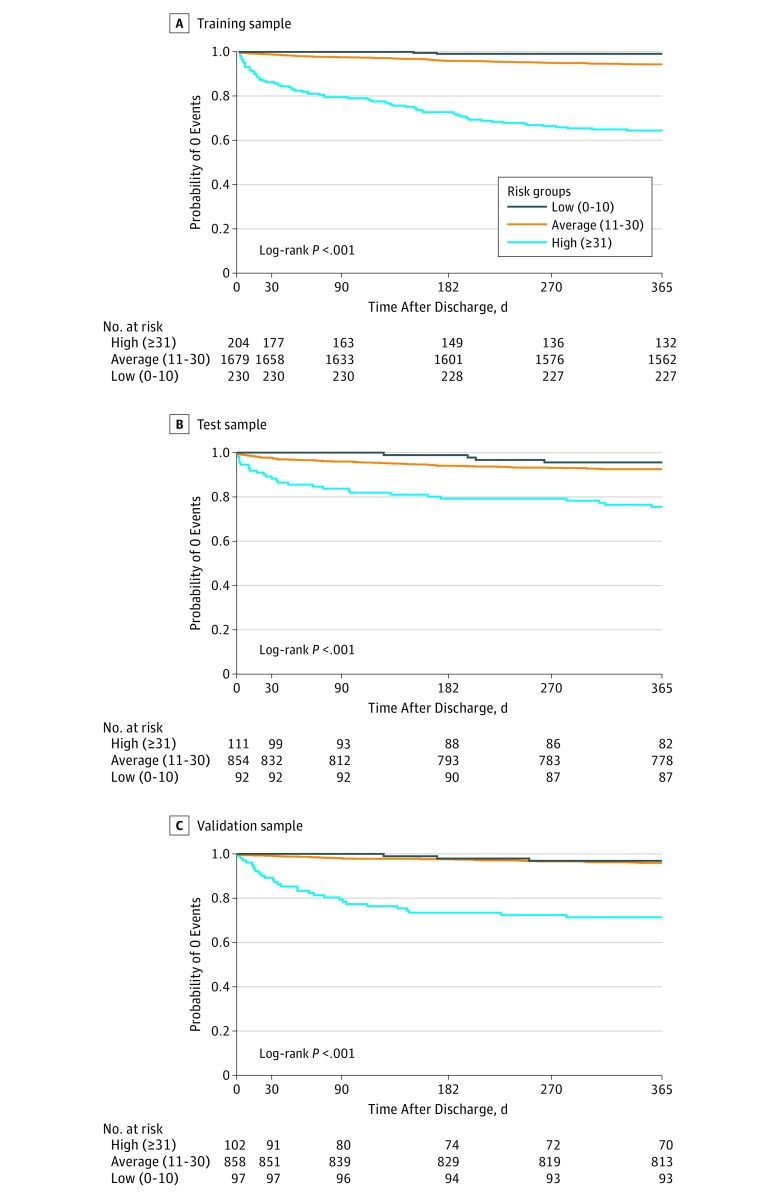
Observed Probability of Being Free From 1-Year Major Cardiovascular Events by Risk Groups

The risk factor–specific points ranged from 2 (hypertension and number of in-hospital complications) to 14 (age ≥85 years) (Table 2). Age, EF, WBC, prior ventricular tachycardia or ventricular fibrillation, prior angina, and heart rate were the top 5 factors with a risk ratio greater than 2.00. The training sample had a mean (SD) risk score of 20.2 (7.9). Scores varied little across the test and validation samples. The mean (SD) score was 20.6 (8.1) for the test sample and 20.4 (7.8) for the validation sample (Figure 1). In the training sample, 11.3%, 81.0%, and 7.7% of patients, respectively, were stratified to the high-, average-, and low-risk groups, with corresponding probabilities of 0.32, 0.06, and 0.01 for 1-year events (Figure 2). Figure 3 shows Kaplan-Meier estimates of the probability of being free from 1-year major cardiovascular events by risk groups. The stratifications for the test and validation samples were not substantially different from those for the training sample (Figure 1, Figure 2, and Figure 3; eTable 2 in the Supplement).

**Table 2.** Final Risk Prediction Model for 1-Year Major Cardiovascular Events After Discharge for Acute Myocardial Infarction Based on Training Sample

Risk Factor	Training Data		
	Regression Coefficient	Hazard Ratio (95% CI)	Points^a^
Age, y			
65-74	0.773	2.17 (1.50-3.14)	6
75-84	1.223	3.40 (2.26-5.10)	9
≥85	1.906	6.73 (2.83-15.96)	14
No college degree	0.525	1.69 (1.00-2.86)	4
No prearrival medical assistance	0.462	1.59 (1.12-2.26)	3
Prior angina	0.716	2.05 (1.17-3.58)	5
Prior acute myocardial infarction	0.494	1.64 (1.07-2.52)	4
Prior ventricular tachycardia or ventricular fibrillation	0.767	2.15 (0.99-4.70)	6
Hypertension	0.267	1.31 (0.94-1.81)	2
Symptoms to admission >4 h	0.360	1.43 (1.03-2.00)	3
Renal dysfunction (blood urea nitrogen >40 mg/dL or creatinine >2.5 mg/dL)	0.487	1.63 (1.18-2.25)	4
Ejection fraction value			
<40%	1.051	2.86 (1.89-4.34)	8
Unmeasured	0.737	2.09 (1.43-3.06)	6
White blood cell count			
6000/μL-12 000/μL	0.493	1.64 (1.08-2.47)	4
>12 000/μL	0.975	2.65 (1.53-4.61)	7
Fasting blood glucose >216 mg/dL	0.599	1.82 (1.13-2.93)	5
Heart rate >90 beats/min	0.702	2.02 (1.43-2.84)	5
Systolic blood pressure <100 mm Hg	0.529	1.70 (1.05-2.74)	4
Each in-hospital complication	0.213	1.24 (1.09-1.40)	2

SI conversion factors: To convert blood urea nitrogen to mmol/L, multiply by 0.357; creatinine to μmol/L, multiply by 88.4; white blood cell count to ×10^9^/L, multiply by 0.001; and blood glucose to mmol/L, multiply by 0.0555.

^a^ Points were calculated by dividing a risk factor’s coefficient by the sum of all coefficients, multiplying by 100, and rounding to the nearest integer.

## Discussion

In this study, we identified 19 risk factors and developed and evaluated a risk model that predicts 1-year major cardiovascular events after discharge for AMI. These factors, selected based on abstracted data from medical records and robust statistical learning algorithms, are easy to collect and are readily available at time of discharge. The risk model and its corresponding risk scores allow clinicians to identify patients who are at heightened risk of 1-year cardiovascular events and might also help patients understand their risk of not only death but also adverse events that could impair their quality of life. The ability to identify individuals with the highest risk of long-term cardiovascular events after AMI may aid in the provision of targeted, intensive, and higher-quality longitudinal care following discharge.

Our study, based on abstracted information and patient interviews, represents a unique, large, and contemporary investigation of the risk factors associated with long-term outcomes following discharge for AMI in China. In contrast to prior studies that focused solely on mortality, we evaluated major cardiovascular events in addition to mortality. Although the overall 1-year mortality rate for patients surviving an AMI is important, many patients experience nonfatal adverse events that can dramatically affect their quality of life. In our sample, 1-year mortality was 2.7% but the rate of 1-year nonfatal cardiovascular events was 5.1%, approximately 89% higher than the mortality rate. Insight regarding the long-term risk of major but nonfatal adverse events is important from a patient perspective, and educating patients regarding their long-term risk might provide an even stronger incentive to follow up and adhere to medications. Through risk stratification, we found that 1 in 10 patients (11.3%) were at high risk of experiencing a major cardiovascular event after discharge, highlighting the importance of identifying these patients at discharge to ensure they receive appropriate, evidence-based, longitudinal care after discharge. Because this high-risk group was small and many events occurred among those at average risk, a population risk reduction approach that focuses on average-risk individuals is also important and may save more lives.

We used the MCMC algorithm to further assess the strength of the risk factors associated with the outcome, in addition to a traditional stepwise approach. One of the challenges in developing a predictive model is balancing the number of factors included in the model and the variance. More factors are desirable for prediction. The MCMC algorithm, which provides a robust Bayesian variable selection approach, allowed us to select a factor based on the marginal posterior probability that the factor should be in the model. Additionally, we used latent class analysis to validate the selected risk factors. The occurrence of major cardiovascular events following discharge is unobservable at discharge and associated with unknown factors after discharge. Such characteristics present a challenge for predictive model development based on conventional regression methods. Latent class analysis is increasingly being used in outcomes research^23,24,25,26,27,28^ and can stratify patients into subgroups based on their risk factors at discharge when outcome information is absent in a training sample. The method is suitable to address at least some of the challenges of a regression approach. By conjoining a traditional Cox regression with latent class analysis, our study used the strengths from both methods and increased the reliability and validity of the selected risk factors. Given the nature of unsupervised learning of the latent class analysis, the discrimination reported by this study is also reasonable.

Our risk factors were identified based on a large patient sample and subsequently tested and validated with high agreement with the training results. Potential factors used for developing a predictive model may influence the strength of the final model in several ways. An ideal risk factor should be unaffected by clinical interpretation, widely accepted, easily collected, and available on admission, during hospitalization, and at discharge. The 19 risk factors identified in this study met these criteria, and many of them have been identified in other studies.^29,30,31^ Some of the selected risk factors are modifiable, providing multiple targets for patient and physician interventions (eg, blood pressure control, lifestyle changes, and appropriate care after discharge) to prevent major cardiovascular events after discharge. One risk factor, the number of in-hospital complications, is related to hospitals and could reflect hospital quality of care. Our data showed that each additional in-hospital complication was associated with a 24% increase in the risk of cardiovascular events after discharge. If a hospital can reduce its complication rate, it could improve its patients’ outcomes after discharge. Although the 19 risk factors were selected based on a Chinese AMI population, factors such as insurance, patient occupation, and hospital length of stay, which may vary across countries, were included in the initial stepwise process but were not identified as the final risk factors. Thus, the 19 risk factors are likely relevant outside of China and could provide clinicians with targets for treatment that may improve prognosis. Validation in additional studies is necessary, but this potential for generalization to other countries underscores the utility of the data presented in this study. Age, EF, WBC, prior ventricular tachycardia or ventricular fibrillation, prior angina, and heart rate were the top 5 factors with a risk ratio greater than 2.00, potentially suggesting that these could be the most clinically important factors.

### Limitations

Our study has several limitations. Our training, test, and validation samples were all drawn from 1 cohort. Although this approach has been used in many prediction studies based on a single cohort,^21,32,33^ the risk factors and model identified in our study should continue to be evaluated and updated as additional data become available. The risk score was calculated based on rounding each risk factor’s coefficient. Although this may result in a loss of information, it provides a simple and easy method for risk prediction in daily clinical practice. To further encourage the use of the score in clinical practice, the score could be implemented as an application for portable devices by adapting a nomogram.^34^

## Conclusions

In conclusion, our 19-factor risk model had good predictive range and was able to identify high-risk patients at the time of discharge. It provides a basis for clinicians to better understand patients’ risk of long-term major cardiovascular events after an AMI and can help clinicians make better-targeted, evidence-based decisions for AMI care following discharge.

## Notes

Supplement. **eAppendix 1.** Clinical Outcome Definitions **eAppendix 2. **Additional Information on Method **eTable 1.** Patient Characteristics by Training, Test, and Validation Samples **eTable 2.** Patient Risk Stratification Based on Risk Score **eFigure 1.** Median Days to 1-Year Major Cardiovascular Events **eFigure 2.** Risk Factors Associated With 1-Year Major Cardiovascular Events Based on the Training Sample **eFigure 3.** Time-Dependent Area Under the ROC Curve Based on Training Sample **eFigure 4.** Probability of 1-Year Major Cardiovascular Events by Deciles in the Training (Left Panel), Test (Middle Panel), and Validation (Right Panel) Samples **eFigure 5.** Observed Versus Predicted Values by Deciles in the Training (Left Panel), Test (Middle Panel), and Validation (Right Panel) Samples **eFigure 6.** Risk Stratification Based on Latent Class Analysis in the Training Sample **eFigure 7.** Association Between Probability of 1-Year Events Based on Regression Coefficients Estimated From the Training Sample and Probability of 1-Year Events Based on Regression Coefficients Estimated From the Test (Left Panel) and Validation (Right Panel) Samples
