# Supplementary material for: Risk Factors Associated With Major Cardiovascular Events 1 Year After Acute Myocardial Infarction
Source: JAMA Netw Open. 2018 Aug 10;1(4):e181079. doi: 10.1001/jamanetworkopen.2018.1079 (PMC6324290; doi:10.1001/jamanetworkopen.2018.1079)
Supplement: Supplement. — eAppendix 1. Clinical Outcome Definitions eAppendix 2. Additional Information on Method eTable 1. Patient Characteristics by Training, Test, and Validation Samples eTable 2. Patient Risk Stratification Based on Risk Score eFigure 1. Median Days to 1-Year Major Cardiovascular Events eFigure 2. Risk Factors Associated With 1-Year Major Cardiovascular Events Based on the Training Sample eFigure 3. Time-Dependent Area Under the ROC Curve Based on Training Sample eFigure 4. Probability of 1-Year Major Cardiovascular Events by Deciles in the Training (Left Panel), Test (Middle Panel), and Validation (Right Panel) Samples eFigure 5. Observed Versus Predicted Values by Deciles in the Training (Left Panel), Test (Middle Panel), and Validation (Right Panel) Samples eFigure 6. Risk Stratification Based on Latent Class Analysis in the Training Sample eFigure 7. Association Between Probability of 1-Year Events Based on Regression Coefficients Estimated From the Training Sample and Probability of 1-Year Events Based on Regression Coefficients Estimated From the Test (Left Panel) and Validation (Right Panel) Samples [file jamanetwopen-1-e181079-s001.pdf]

Supplementary Online Content

Wang Y, Li J, Zheng X, et al. Risk Factors Associated With Major Cardiovascular Events 1 Year After Acute Myocardial Infarction. *JAMA Netw Open.* 2018;1(4):e181079. doi:10.1001/jamanetworkopen.2018.1079

eAppendix 1. Clinical Outcome Definitions
eAppendix 2. Additional Information on Method
eTable 1. Patient Characteristics by Training, Test, and Validation Samples
eTable 2. Patient Risk Stratification Based on Risk Score
eFigure 1. Median Days to 1-Year Major Cardiovascular Events
eFigure 2. Risk Factors Associated With 1-Year Major Cardiovascular Events Based on the Training Sample
eFigure 3. Time-Dependent Area Under the ROC Curve Based on Training Sample
eFigure 4. Probability of 1-Year Major Cardiovascular Events by Deciles in the Training (Left Panel), Test (Middle Panel), and Validation (Right Panel) Samples
eFigure 5. Observed Versus Predicted Values by Deciles in the Training (Left Panel), Test (Middle Panel), and Validation (Right Panel) Samples
eFigure 6. Risk Stratification Based on Latent Class Analysis in the Training Sample
eFigure 7. Association Between Probability of 1-Year Events Based on Regression Coefficients Estimated From the Training Sample and Probability of 1-Year Events Based on Regression Coefficients Estimated From the Test (Left Panel) and Validation (Right Panel) Samples

This supplementary material has been provided by the authors to give readers additional information about their work.

© 2018 Wang Y et al. *JAMA Network Open.*

Online Supplement

eAppendix 1. Clinical outcome definitions

Recurrent myocardial infarction

The term Myocardial Infarction (MI) should only be used when there is evidence of myocardial necrosis consistent with myocardial ischemia in an appropriate clinical context. In general, the diagnosis of MI requires the following to be true:

	i. Cardiac necrosis	
	- Cardiac biomarkers	Cardiac biomarker results compatible with acute myocardial necrosis
	or - Autopsy	Myocardial necrosis/scar or coronary thrombus of an age consistent with the clinical presentation
AND	ii. Other supporting information:	
	- Presentation	i. Symptoms of ischemia; or<br>ii. Death
	or - ECG	i. Ischemic changes (not known to be old); or<br>ii. Development of pathological Q waves
	or - Imaging	i. New myocardial defect; or<br>ii. Acute coronary occlusion
AND	iii. Exclusions:	No other likely diagnosis

Hospitalized heart failure

Hospitalized heart failure is defined as an event where the patient is admitted to the hospital with a primary diagnosis of heart failure and the length of stay is at least 24 hours (or extends over a calendar date if the hospital admission and discharge times are unavailable), exhibits new or worsening symptoms of heart failure on presentation, has objective evidence of new or worsening heart failure, and receives treatment (initiation or intensification) specifically for heart failure.

© 2018 Wang Y et al. *JAMA Network Open.*

Ischemic/hemorrhagic stroke

Ischemic stroke is defined as an acute symptomatic episode of focal or global neurological dysfunction caused by brain, spinal, or retinal vascular injury as a result of infarction. Hemorrhagic stroke occurs when a blood vessel in the brain breaks, leaking blood into the surrounding tissue.

Vascular death and all-cause death

Vascular death includes death due to cardiovascular diseases (e.g., AMI, heart failure, stroke, coronary revascularization, hospitalized unstable angina). All-cause death is defined as any death regardless of cause.

Data collection

All post-discharge outcome information was collected through follow-up interviews at one, six, and 12 months after discharge. During the follow-up period, trained staff interviewed patients and systematically sought information on all clinical events, including recurrent AMI, angina, stroke, heart failure, transient ischemic attack, repeated angiography procedures, revascularization, bleeding, and rehospitalizations. If a patient died during follow-up, the death information was obtained from the patient’s family or physician. If a patient was unable to attend an onsite interview, information was obtained by telephone through direct correspondence with the patient, patient's family members, or patient's physician.

© 2018 Wang Y et al. *JAMA Network Open.*

eAppendix 2. Additional Information on Method

We further validated the selected risk factors by comparing their performance in the training sample with their performance in the test and validation samples. We first calculated two probabilities of 1-year events for each patient in the test sample; one was based on regression coefficients estimated from the training model, and one was based on regression coefficients estimated from the test model. If the selected risk factors are stable, these two probabilities shall be highly associated. We also evaluated the selected risk factors by comparing their performance in the training model with their performance in the test model. Specifically, we fit a Cox model with the probability of 1-year events based on regression coefficients estimated from the training sample and assessed the model's performance indicators, described in the main text, between the training and test models. We repeated these processes for the validation sample.

© 2018 Wang Y et al. *JAMA Network Open.*

eTable 1. Patient characteristics by training, test, and validation samples

Characteristic	Aggregate	Training	Test	Validation
**Number of patients**	4227	2113	1057	1057
**Demographics**				
Female, # (%)	994 (23.5)	498 (23.6)	244 (23.1)	252 (23.8)
Age ≥65 years, # (%)	1655 (39.2)	815 (38.6)	392 (37.1)	448 (42.4)
Mean age (SD)				
Worker, # (%)	1256 (29.7)	605 (28.6)	319 (30.2)	332 (31.4)
Farmer, # (%)	948 (22.4)	464 (22.0)	238 (22.5)	246 (23.3)
No insurance, # (%)	109 (2.6)	47 (2.2)	31 (2.9)	31 (2.9)
Married, # (%)	3596 (85.1)	1805 (85.4)	896 (84.8)	895 (84.7)
Without a college degree, # (%)	3661 (86.6)	1822 (86.2)	918 (86.9)	921 (87.1)
**Medical history or comorbidity**				
Prior angina, # (%)	166 (3.9)	86 (4.1)	43 (4.1)	37 (3.5)
Prior acute myocardial infarction, # (%)	332 (7.9)	164 (7.8)	83 (7.9)	85 (8.0)
Prior percutaneous coronary intervention, # (%)	277 (6.6)	143 (6.8)	60 (5.7)	74 (7.0)
Prior coronary artery bypass grafting, # (%)	6 (0.1)	2 (0.1)	2 (0.2)	2 (0.2)
Prior coronary heart disease, # (%)	1798 (42.5)	904 (42.8)	430 (40.7)	464 (43.9)
Prior ventricular tachycardia/ventricular fibrillation, # (%)	100 (2.4)	46 (2.2)	23 (2.2)	31 (2.9)
Prior atrial fibrillation, # (%)	123 (2.9)	57 (2.7)	35 (3.3)	31 (2.9)
Prior heart failure, # (%)	1084 (25.6)	544 (25.7)	261 (24.7)	279 (26.4)
Diabetes mellitus, # (%)	1004 (23.8)	501 (23.7)	237 (22.4)	266 (25.2)
Dyslipidemia, # (%)	1290 (30.5)	654 (31.0)	330 (31.2)	306 (28.9)
Chronic renal failure, # (%)	103 (2.4)	47 (2.2)	30 (2.8)	26 (2.5)
Family history of coronary heart disease, # (%)	457 (10.8)	233 (11.0)	109 (10.3)	115 (10.9)
Hypertension, # (%)	2358 (55.8)	1164 (55.1)	590 (55.8)	604 (57.1)
Major surgery within the past 4 weeks, # (%)	83 (2.0)	50 (2.4)	10 (0.9)	23 (2.2)
Prior ischemic stroke, # (%)	31 (0.7)	17 (0.8)	7 (0.7)	7 (0.7)

© 2018 Wang Y et al. *JAMA Network Open.*

Peripheral artery disease, # (%)	30 (0.7)	15 (0.7)	6 (0.6)	9 (0.9)
Cancer, # (%)	26 (0.6)	14 (0.7)	4 (0.4)	8 (0.8)
Thyroid disease, # (%)	48 (1.1)	36 (1.7)	8 (0.8)	4 (0.4)
Prior pneumonia, # (%)	461 (10.9)	235 (11.1)	111 (10.5)	115 (10.9)
Anemia, # (%)	581 (13.7)	290 (13.7)	146 (13.8)	145 (13.7)
Liver disease, # (%)	65 (1.5)	29 (1.4)	17 (1.6)	19 (1.8)
Parental history of acute myocardial infarction, percutaneous coronary intervention, or coronary artery bypass, # (%)	454 (10.7)	231 (10.9)	108 (10.2)	115 (10.9)
**Characteristic**	**Aggregate**	**Training**	**Test**	**Validation**
Never smoked, # (%)	1188 (28.1)	601 (28.4)	290 (27.4)	297 (28.1)
**Coexisting conditions at presentation**				
Symptoms-to-admission >4 hours, # (%)	2438 (57.7)	1227 (58.1)	617 (58.4)	594 (56.2)
Current smoking, # (%)	2441 (57.7)	1203 (56.9)	620 (58.7)	618 (58.5)
Killip class 3 or 4, # (%)	186 (4.4)	101 (4.8)	48 (4.5)	37 (3.5)
Renal dysfunction (blood urea nitrogen >40 mg/dL or creatinine >2.5 mg/dL) , # (%)	844 (20.0)	401 (19.0)	239 (22.6)	204 (19.3)
No ST-elevation, # (%)	842 (19.9)	425 (20.1)	203 (19.2)	214 (20.2)
ST-depression, # (%)	368 (8.7)	180 (8.5)	95 (9.0)	93 (8.8)
Acute inferior myocardial infarction, # (%)	3422 (81.0)	1701 (80.5)	860 (81.4)	861 (81.5)
Acute anterior myocardial infarction, # (%)	1192 (28.2)	590 (27.9)	310 (29.3)	292 (27.6)
Heart failure, # (%)	1077 (25.5)	540 (25.6)	260 (24.6)	277 (26.2)
Duration of ischemia symptoms >20 minutes, # (%)	3065 (72.5)	1541 (72.9)	745 (70.5)	779 (73.7)
Ejection fraction value <40%, # (%)	310 (7.3)	149 (7.1)	86 (8.1)	75 (7.1)
Ejection fraction value unmeasured, # (%)	598 (14.1)	293 (13.9)	159 (15.0)	146 (13.8)
White blood cell count in 6-12 $\times 10^3$/µL, # (%)	2882 (68.2)	1439 (68.1)	724 (68.5)	719 (68.0)
White blood cell count >12 $\times 10^3$/µL, #	351 (8.3)	191 (9.0)	85 (8.0)	75 (7.1)

© 2018 Wang Y et al. *JAMA Network Open.*

(%)				
Fasting blood-glucose >216 mg/dL, # (%)	254 (6.0)	125 (5.9)	68 (6.4)	61 (5.8)
Heart rate >90 beats per minute, # (%)	598 (14.1)	307 (14.5)	139 (13.2)	152 (14.4)
Mean admission temperature (SD), °C	36.4 (0.3)	36.4 (0.3)	36.4 (0.3)	36.4 (0.3)
One vessel disease, # (%)	1352 (32.0)	672 (31.8)	322 (30.5)	358 (33.9)
Two vessel disease, # (%)	1186 (28.1)	590 (27.9)	292 (27.6)	304 (28.8)
Three vessel disease, # (%)	660 (15.6)	340 (16.1)	163 (15.4)	157 (14.9)
Systolic blood pressure >140 mm Hg, # (%)	1244 (29.4)	625 (29.6)	319 (30.2)	300 (28.4)
Mean systolic blood pressure (SD), mm Hg	132.0 (42.9)	132.3 (44.9)	131.5 (36.5)	131.6 (44.7)
**In-hospital complications**				
Recurrent angina, # (%)	1147 (27.1)	566 (26.8)	299 (28.3)	282 (26.7)
Atrial fibrillation or flutter, # (%)	135 (3.2)	65 (3.1)	40 (3.8)	30 (2.8)
Cardiopulmonary resuscitation, # (%)	67 (1.6)	30 (1.4)	17 (1.6)	20 (1.9)
Ventricular tachycardia, # (%)	207 (4.9)	97 (4.6)	59 (5.6)	51 (4.8)
Ventricular fibrillation, # (%)	113 (2.7)	53 (2.5)	24 (2.3)	36 (3.4)
Heart failure (new onset), # (%)	350 (8.3)	184 (8.7)	88 (8.3)	78 (7.4)
Infection, # (%)	635 (15.0)	333 (15.8)	163 (15.4)	139 (13.2)
Stroke, # (%)	138 (3.3)	52 (2.5)	41 (3.9)	45 (4.3)
Major bleeding, # (%)	423 (10.0)	212 (10.0)	109 (10.3)	102 (9.6)
Recurrent acute myocardial infarction, # (%)	44 (1.0)	22 (1.0)	10 (0.9)	12 (1.1)
**Characteristic**	**Aggregate**	**Training**	**Test**	**Validation**
**Treatment within 24 hours of admission**				
Without pre-arrival medical assistance, # (%)	2655 (62.8)	1296 (61.3)	690 (65.3)	669 (63.3)
Aspirin prior to arrival, # (%)	578 (13.7)	297 (14.1)	132 (12.5)	149 (14.1)
Aspirin in the emergency, # (%)	482 (11.4)	259 (12.3)	113 (10.7)	110 (10.4)
Clopidogrel in the emergency room, # (%)	450 (10.6)	230 (10.9)	120 (11.4)	100 (9.5)
Heparin in the emergency room, # (%)	53 (1.3)	25 (1.2)	14 (1.3)	14 (1.3)
Aspirin, # (%)	3398 (80.4)	1708 (80.8)	846 (80.0)	844 (79.8)
Heparin, # (%)	3765 (89.1)	1898	943 (89.2)	924 (87.4)

© 2018 Wang Y et al. *JAMA Network Open.*

		(89.8)		
Beta-blocker, # (%)	2098 (49.6)	1026 (48.6)	539 (51.0)	533 (50.4)
Clopidogrel, # (%)	3843 (90.9)	1928 (91.2)	961 (90.9)	954 (90.3)
Angiotensin-converting enzyme inhibitor/angiotensin-receptor blocker, # (%)	1990 (47.1)	1000 (47.3)	494 (46.7)	496 (46.9)
**In-hospital treatment**				
Thrombolysis (prior to admission/ER/in-hospital), # (%)	587 (13.9)	304 (14.4)	152 (14.4)	131 (12.4)
Reperfusion, # (%)	1872 (44.3)	935 (44.2)	465 (44.0)	472 (44.7)
Complete revascularization, # (%)	190 (4.5)	91 (4.3)	53 (5.0)	46 (4.4)
Partial revascularization, # (%)	1353 (32.0)	676 (32.0)	324 (30.7)	353 (33.4)
Heparin, # (%)	4007 (94.8)	2013 (95.3)	1006 (95.2)	988 (93.5)
Aspirin, # (%)	3855 (91.2)	1922 (91.0)	968 (91.6)	965 (91.3)
Beta-blocker, # (%)	3439 (81.4)	1693 (80.1)	874 (82.7)	872 (82.5)
Clopidogrel, # (%)	4063 (96.1)	2028 (96.0)	1022 (96.7)	1013 (95.8)
Angiotensin-converting enzyme inhibitor/angiotensin-receptor blocker, # (%)	3075 (72.7)	1543 (73.0)	782 (74.0)	750 (71.0)
Primary percutaneous coronary intervention, # (%)	1256 (29.7)	622 (29.4)	307 (29.0)	327 (30.9)
Coronary artery bypass grafting, # (%)	32 (0.8)	12 (0.6)	6 (0.6)	14 (1.3)
Statin, # (%)	4167 (98.6)	2085 (98.7)	1038 (98.2)	1044 (98.8)
**Discharge medications**				
Aspirin, # (%)	3063 (72.5)	1522 (72.0)	764 (72.3)	777 (73.5)
Beta-blocker, # (%)	2143 (50.7)	1058 (50.1)	547 (51.8)	538 (50.9)
Clopidogrel, # (%)	3115 (73.7)	1548 (73.3)	788 (74.6)	779 (73.7)

© 2018 Wang Y et al. *JAMA Network Open.*

Angiotensin-converting enzyme inhibitor / angiotensin- receptor blocker, # (%)	2051 (48.5)	1038 (49.1)	522 (49.4)	491 (46.5)
Statin, # (%)	3182 (75.3)	1589 (75.2)	792 (74.9)	801 (75.8)
Suggestions-smoking cession, # (%)	620 (14.7)	315 (14.9)	152 (14.4)	153 (14.5)
**Median length of stay**				
Median (inter-quartile range), days	11(8, 14)	11(8, 14)	11(8, 14)	11(8, 14)

© 2018 Wang Y et al. *JAMA Network Open.*

eTable 2. Patient risk stratification based on risk score

Risk group	Training (n=2,113)		Test (n=1057)		Validation (n=1057)	
	Patients, # (%)	Probability of 1-year events, mean (SD)	Patients, # (%)	Probability of 1-year events, mean (SD)	Patients, # (%)	Probability of 1-year events, mean (SD)
High (risk score 31+)	238 (11.3)	0.32 (0.14)	128 (12.1)	0.34 (0.18)	124 (11.7)	0.32 (0.14)
Middle (risk score 11-30)	1713 (81.0)	0.06 (0.04)	865 (81.8)	0.06 (0.04)	857 (81.1)	0.06 (0.04)
Low (risk score 0-10)	163 (7.7)	0.01 (<0.01)	64 (6.1)	0.01 (<0.01)	76 (7.2)	0.01 (<0.01)

© 2018 Wang Y et al. *JAMA Network Open.*

eFigure 1. Median days to 1-year major cardiovascular events

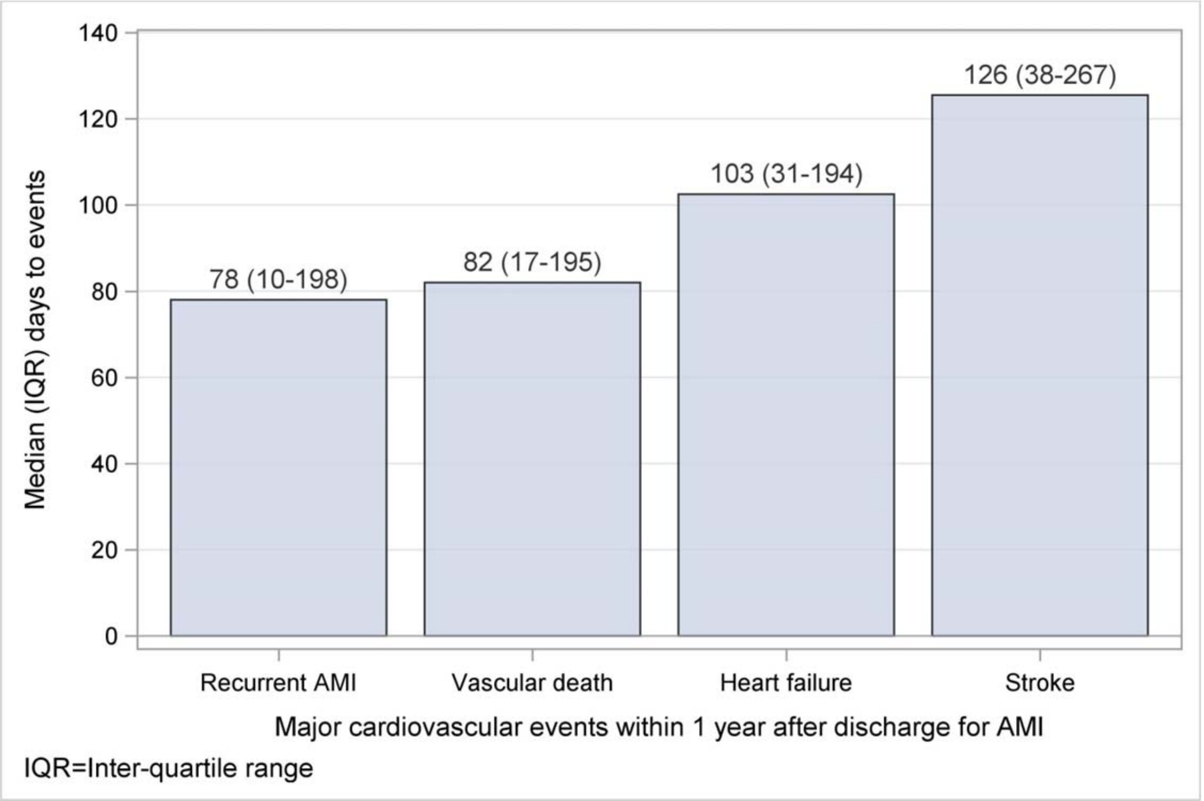

© 2018 Wang Y et al. *JAMA Network Open.*

eFigure 2. Risk factors associated with 1-year major cardiovascular events based on the training sample

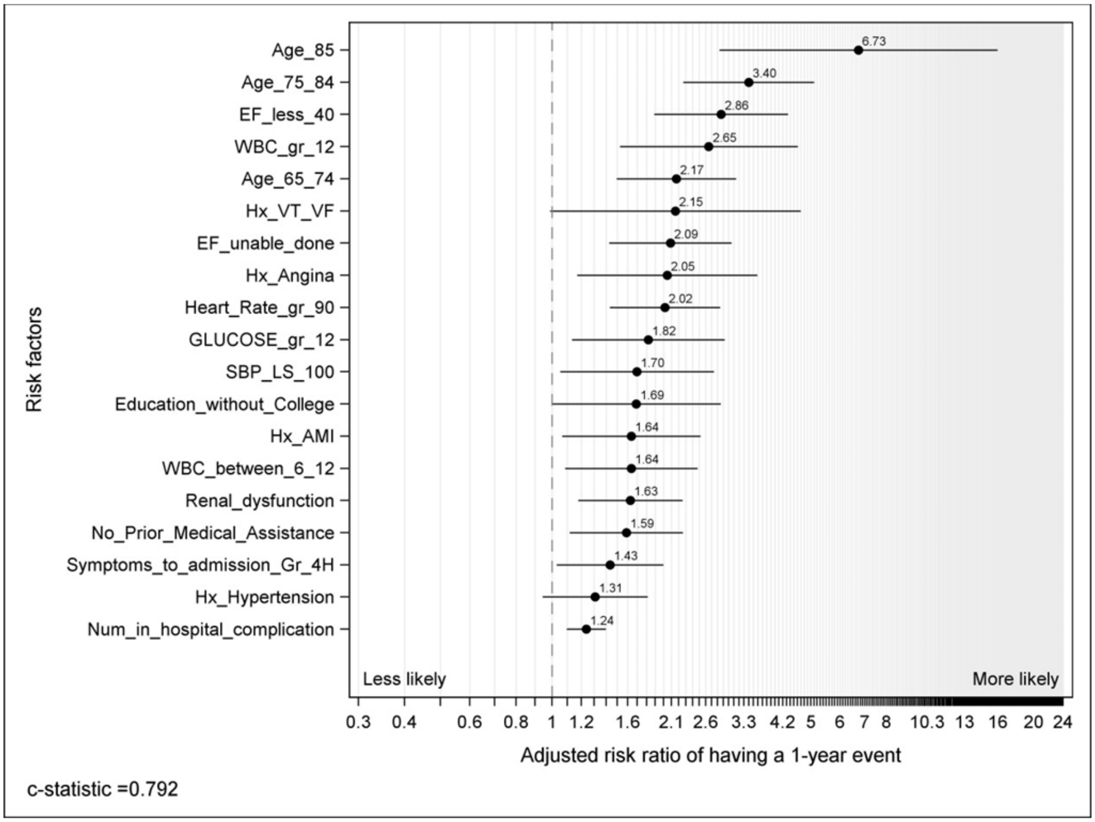

© 2018 Wang Y et al. *JAMA Network Open.*

eFigure 3. Time-dependent area under the ROC curve based on training sample

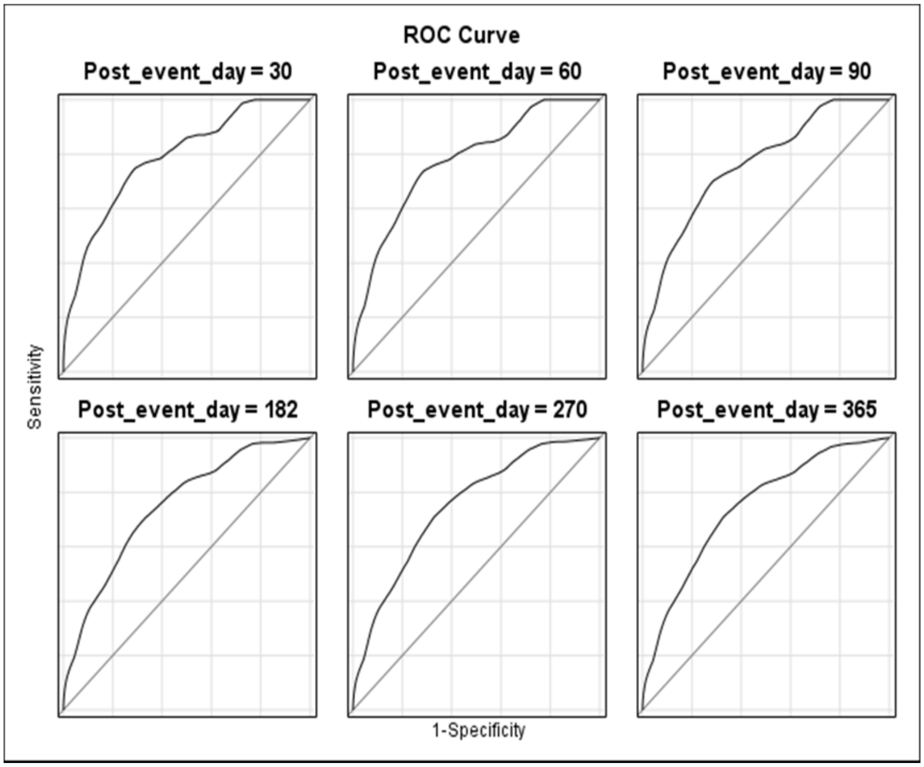

© 2018 Wang Y et al. *JAMA Network Open.*

eFigure 4. Probability of 1-year major cardiovascular events by deciles in the training (left panel), test (middle panel), and validation (right panel) samples.

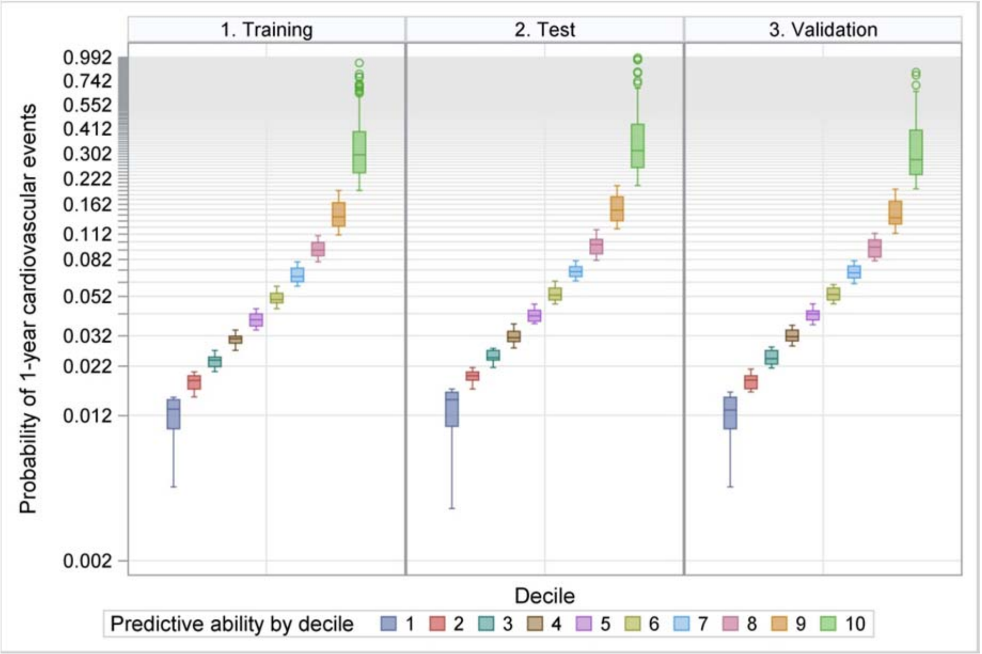

© 2018 Wang Y et al. *JAMA Network Open.*

eFigure 5. Observed versus predicted values by deciles in the training (left panel), test (middle panel), and validation (right panel) samples.

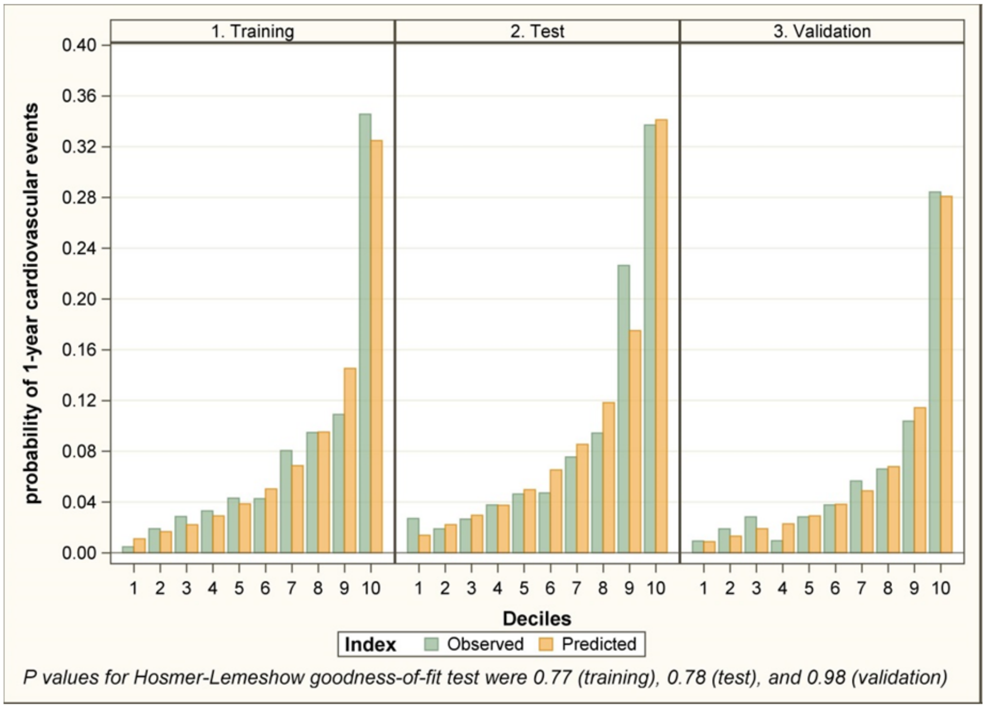

© 2018 Wang Y et al. *JAMA Network Open.*

eFigure 6. Risk stratification based on latent class analysis in the training sample

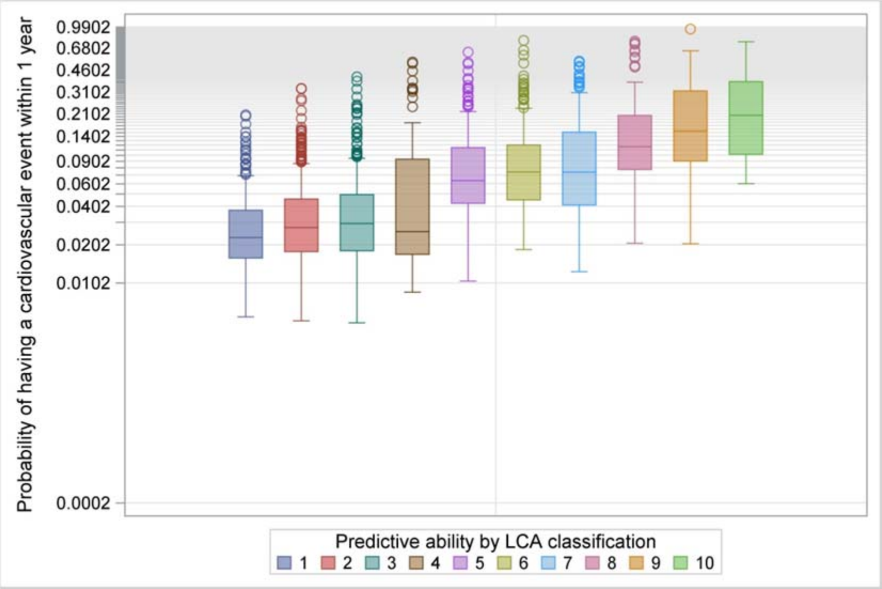

© 2018 Wang Y et al. *JAMA Network Open.*

eFigure 7. Association between probability of 1-year events based on regression coefficients estimated from the training sample and probability of 1-year events based on regression coefficients estimated from the test (left panel) and validation (right panel) samples

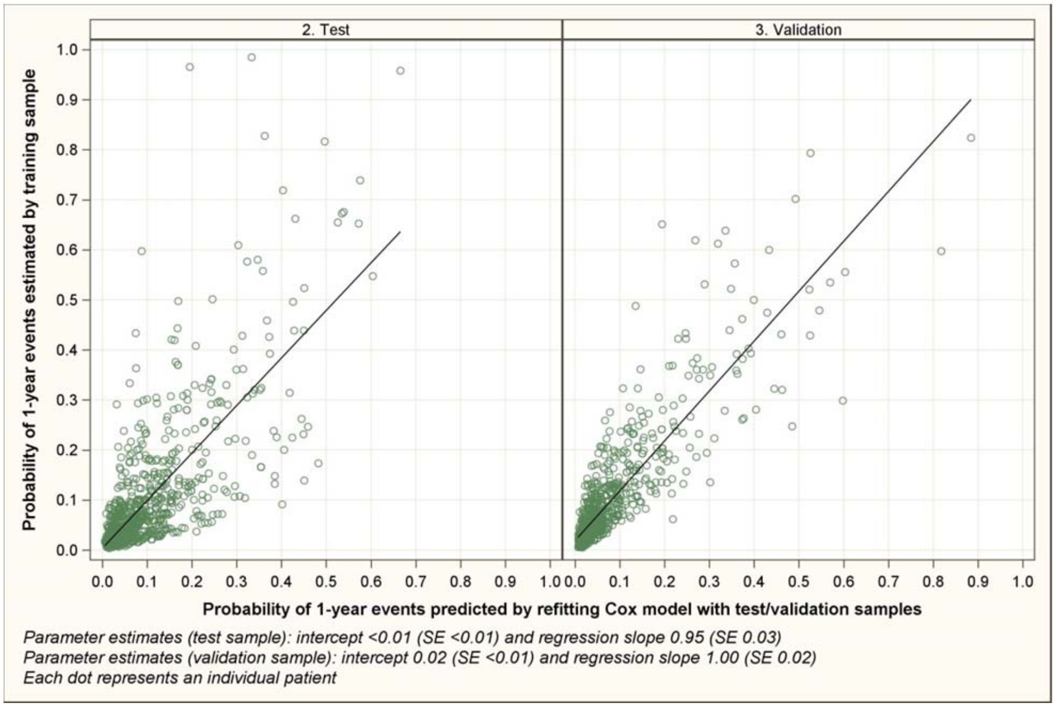

© 2018 Wang Y et al. *JAMA Network Open.*
